# Synthesis and Curing of Tetra-Component Phenolic Resin as a Candidate Resin for Further Investigation in Photoresist Formulations

**DOI:** 10.3390/ma19204295

**Published:** 2026-10-12

**Authors:** Xiaochuan Wang, Zhili Xu, Lichen Lin, Qiang Lin, Fengbing Liang, Dexin Feng

**Affiliations:** 1 CAS Key Laboratory of Biobased Materials, Qingdao Institute of Bioenergy and Bioprocess Technology, Chinese Academy of Sciences, Qingdao 266101, China; wangxiaochuan@qibebt.ac.cn; 2 Shandong Energy Institute, Qingdao 266101, China; 3 Qingdao New Energy Shandong Laboratory, Qingdao 266101, China; 4 Jiangsu Rebo New Materials Technology Co., Ltd., Lianyungang 222200, China

**Keywords:** photoresist, phenolic resin, hexamethoxymethyl melamine, condensation reactions, crosslinking

## Abstract

The paper describes the synthesis and properties of a tetra-component phenolic resin as a candidate resin for further investigation in photoresist formulations, and its oligomer was synthesized via addition, condensation, and polycondensation reactions. Characterization was conducted using infrared spectroscopy, nuclear magnetic resonance, and gel permeation chromatography. The tetra-component phenolic resin has a weight-average molecular weight of 6376 g/mol and an average alkali solubility rate of 159 ± 2.16 Å/s. Acid-catalyzed crosslinking of HMMM and phenolic resin were used to simulate the crosslinking process of a novolac negative photoresist induced by photoacid. The results indicate that this tetra-component phenolic resin preliminarily meets the requirements for photoresist film formation and could have the opportunity to be used as a candidate resin for further investigation in photoresist formulations.

## 1. Introduction

Photolithography technology is an important process in today’s electronics industry [1,2,3]. The principle of photolithography originated from photographic plate making in printing technology. As a special chemical material, photoresist was initially mainly used for industrial printing [4,5]. With the development of technology, it has begun to be applied more in the field of the electronics industry. In semiconductor manufacturing and liquid crystal display (LCD) production, photoresist plays an important role as a key photosensitive material [6,7,8,9]. So far, photoresist has become one of the key materials for micro pattern processing in the electronic field. Without photoresist participating in the early stage of semiconductor integrated circuit chip production, it is difficult to have innovative products in the future [10,11,12,13]. Electronic-grade phenolic resin is a key raw material for semiconductor packaging, copper clad laminates, photoresists, etc. [14]. Electronic-grade phenolic resin, as a basic polymer, not only regulates the characteristics of photoresist, but also plays a core role in these key electronic industries [15,16,17,18,19].

The i-line photoresist is composed of linear phenolic resin (novolac) and diazonaphthoquinone (DNQ). Novolac serves as the film-forming material, while DNQ endows the system with photosensitive properties. The patterning mechanism of novolac–DNQ photoresist lies in the decomposition of DNQ after exposure, which generates indene carboxylic acid that is soluble in an alkaline developing solution, while in non-exposed areas, DNQ inhibits the dissolution of the resin through hydrogen bonds and azo coupling with phenolic resin [20,21,22]. Novolac is generally a condensation product of m-cresol, p-cresol, and formaldehyde [23,24]. Currently, numerous products, including power semiconductor devices, MEMS and display panels, are manufactured using the novolac–DNQ system for lithography. At present, power semiconductor devices, MEMS, and display panels are predominantly manufactured using photoresists based on the novolac–DNQ system. As the performance of DNQ-based photoresists continues to improve, the demands on the resolution and thermal resistance of i-line photoresists have become increasingly stringent. Therefore, the development of high-resolution, high-thermal-resistance phenolic resins for i-line photoresists is of great significance [25,26,27,28,29].

Novolac is generally synthesized by one-step condensation of meta-cresol, para-cresol, and formaldehyde [19]. To pursue higher resolution, we used a two-step method to prepare novolac [23]. Therefore, a tetra-component phenolic resin was synthesized via addition, condensation, and polycondensation reactions using oxalic acid as a catalyst. The four monomers employed were 2,6-bis(hydroxymethyl)-4-methyl-phenol, resorcinol, m-cresol, and 3,5-dimethylphenol. According to literature reports, the three-component phenolic resin containing p-cresol, 3,5-dicresol, and resorcinol has a higher resolution and thermal deformation temperature than the two-component phenolic resin without resorcinol. I-line exposure can obtain a positive shape with a resolution of 0.5–0.7 μm [30]. Introducing xylenol for end capping in the polymerization system can control the relative molecular weight and solubility of phenolic resin, resulting in a resolution of a 0.25 μm positive i-line photoresist [31,32]. Introduce a 2,6-bis(hydroxymethyl)-4-methylphenol monomer to promote the formation of an ortho-ortho’ structure; introduce a 3,5-dimethylphenol monomer to adjust the alkali dissolution rate. The phenolic resin exhibits a novel structure and can be used as a candidate resin for further investigation in photoresist formulations. The resulting resin structure was characterized by Fourier transform infrared (FTIR) spectroscopy, nuclear magnetic resonance (NMR) spectroscopy, and gel permeation chromatography (GPC). Additionally, acid-catalyzed crosslinking of hexamethoxymethylmelamine (HMMM) and phenolic resin were used to simulate the crosslinking process of novolac negative photoresist induced by photoacid.

## 2. Experimental Details

### 2.1. Reagents and Chemicals

Melamine, 3,5-dimethylphenol, resorcinol, diethylene glycol dimethyl ether, and m-cresol (analytical grade) were purchased from Shanghai Macklin Biochemical Technology Co., Ltd. (Shanghai, China). Oxalic acid dihydrate (analytical grade) was obtained from Fuchen (Tianjin) Chemical Reagent Co., Ltd. (Tianjin, China). Propylene glycol monomethyl ether acetate (PGMEA) and NaHCO_3_ (analytical grade) were supplied by Energy Chemical (Anhui Zesheng Technology Co., Ltd., Hefei, China). Dichloromethane (analytical grade) was purchased from Chengdu Kelong Chemical Reagent Factory (Chengdu, China). p-Toluene sulfonic acid monohydrate and sodium hydroxide (analytical grade) were purchased from Shanghai Titan Technology Co., Ltd. (Shanghai, China). An analytical-grade 37% formaldehyde solution was supplied by Guangzhou Chemical Reagent Factory (Guangzhou, China). Nitric acid (analytical grade) was supplied by Tianjin Chemical Reagent Factory. Ethyl acetate (analytical grade) was obtained from Chengdu Kelong Chemical Reagent Factory.

### 2.2. Instruments

The following instruments were used for characterization and fabrication: FTIR spectra were recorded on a Nicolet Summit X spectrometer (Thermo Fisher Scientific, Shanghai, China). NMR spectra were obtained using a JNM-ECZ400S spectrometer (JEOL Ltd., Tokyo, Japan). GPC analysis was performed on a GPC-20A system (Shimadzu Corporation, Shanghai, China). DSC analyses were conducted with a NETZSCH DSC200F3 (NETZSCH Corporation, Selb, Germany). Film thickness was measured with an F20 thin-film measurement instrument (Filmetrics Inc., San Diego, CA, USA). Spin-coating was carried out using a WS-650Mz-23NPPB spin coater (Laurell Technologies Corporation, North Wales, PA, USA).

### 2.3. Synthesis of 2,6-Bis(hydroxymethyl)-4-Methylphenol (Scheme 1) [8]

In a 250 mL three-neck flask equipped with a mechanical stirrer, 54.00 g (0.5 mol) of p-cresol and 100.00 g of a 20% aqueous solution of NaOH were added, stirred evenly until dissolved, and then 89.20 g of a 37% aqueous solution of formaldehyde was added dropwise. The reaction mixture was stirred continuously at 60 °C for 30 min. After adding 200 mL of distilled water and 40 mL of concentrated hydrochloric acid, a large amount of solid precipitated. After filtration, washing with distilled water, and drying, 80.00 g of light-yellow powder was obtained (yield 95%). The masses of the reagents were measured using an analytical balance with a readability of 0.01 g, and the reaction temperature was maintained within ±1 °C of the set temperature.

**Scheme 1:**
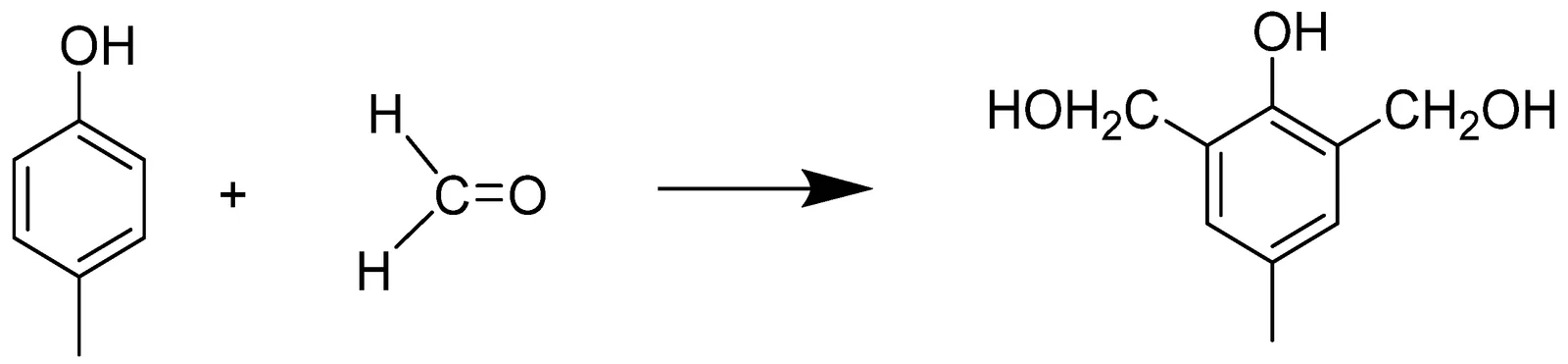
The synthetic route of 2,6-bis(hydroxymethyl)-4-methylphenol.

### 2.4. Synthesis of Tetra-Component Phenolic Resin (Scheme 2)

A 250 mL three-necked flask, fitted with a mechanical stirrer and a reflux condenser, was charged with 2,6-bis(hydroxymethyl)-4-methylphenol (40.40 g, 0.24 mol), 3,5-dimethylphenol (22.00 g, 0.18 mol), resorcinol (2.20 g, 0.02 mol), m-cresol (21.60 g, 0.20 mol), oxalic acid dihydrate (1.20 g), distilled water (10.00 g), and diethylene glycol dimethyl ether (100.00 g). The mixture was stirred and heated until homogeneous, after which the reaction was conducted at 100 °C under reflux for 18 h. Upon completion, the solvent was removed under reduced pressure, and the hot residue was poured out at 180 °C. The masses of the reagents were measured using an analytical balance with a readability of 0.01 g, and the reaction temperature was maintained within ±1 °C of the set temperature. Trace residual solvents may cause certain deviations in subsequent testing.

**Scheme 2:**
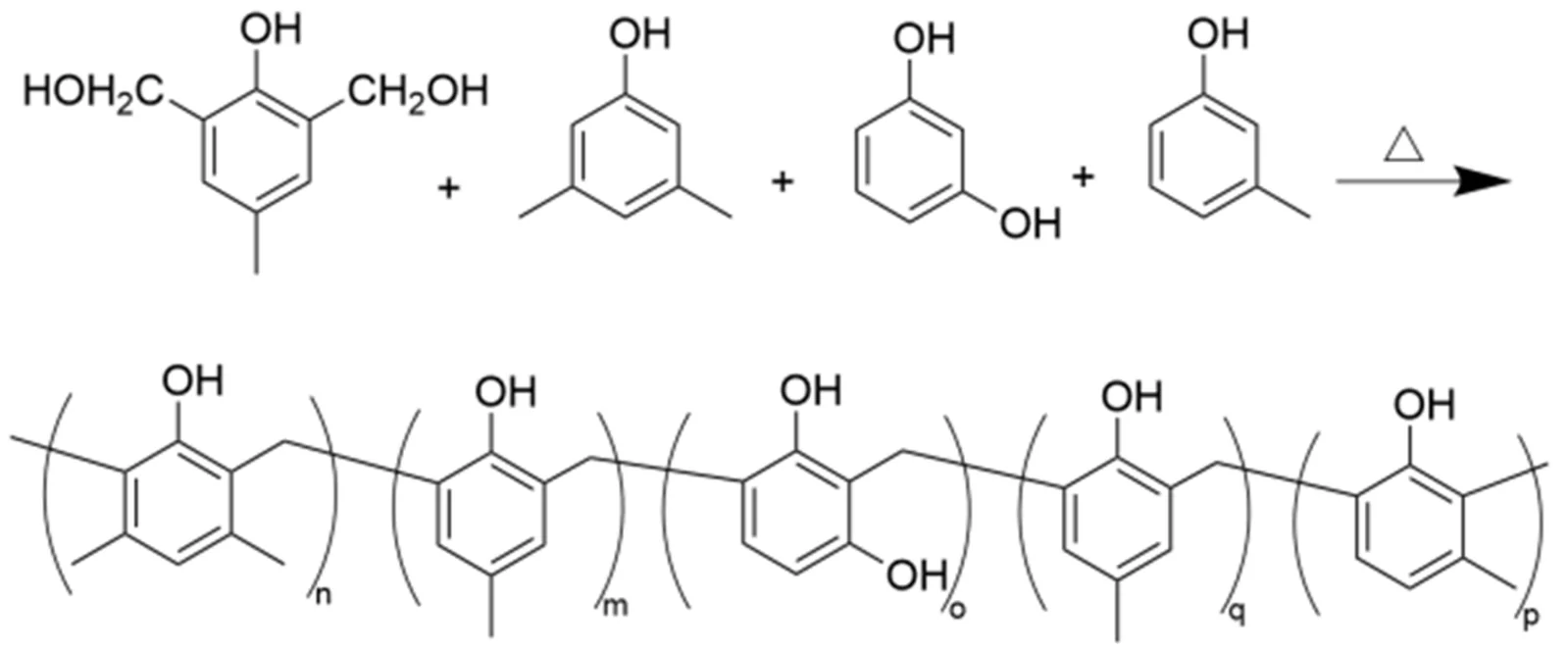
The synthetic route of tetra-component phenolic resin.

Evaluation of the selected reaction conditions which affect the synthesis of tetra-component phenolic resin is summarized in Table 1. The highest softening point is 135 °C, which belongs to entry 1: the evaluation of the impact of the selected reaction conditions.

### 2.5. Synthesis of HMMM (Scheme 3)

In a three-neck flask equipped with a mechanical stirrer, 162.00 g of 37% formaldehyde solution, 25.20 g of melamine, and 1.00 g of sodium hydroxide were added. The mixture was heated to 65 °C, and the system became clear and transparent, then quickly solidified into a hard block. After washing six times with 200 mL of distilled water, the mixture was dried at 75 °C for 12 h to obtain 60.20 g of HMMM (yield 82%). The masses of the reagents were measured using an analytical balance with a readability of 0.01 g, and the reaction temperature was maintained within ±1 °C of the set temperature.

**Scheme 3:**
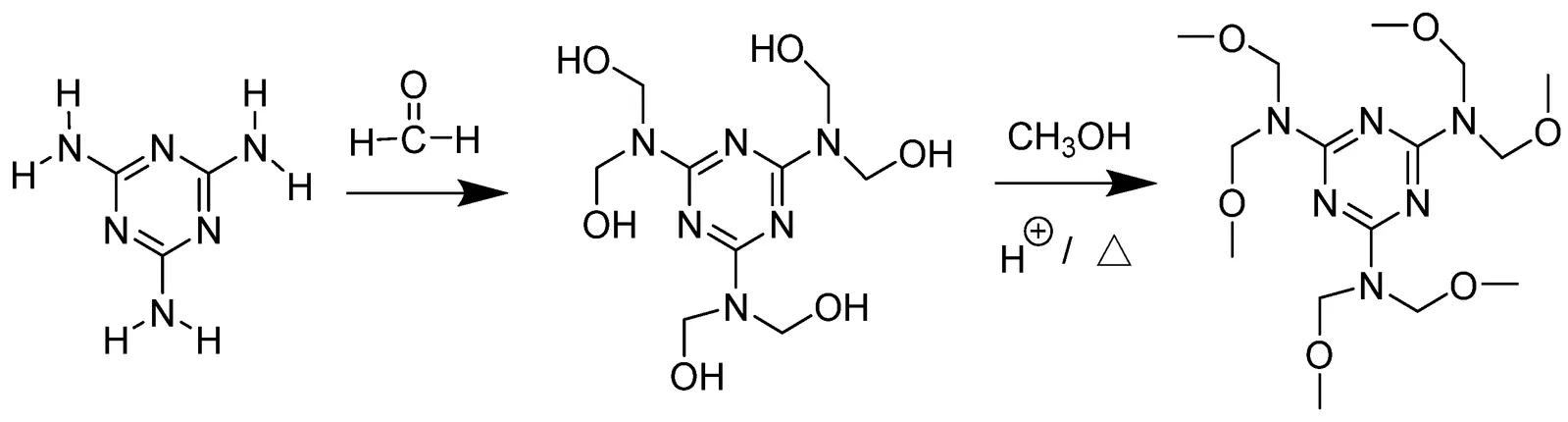
The synthetic route of HMMM.

In a three-neck flask equipped with a mechanical stirrer, 23.50 g of HMMM, 95.00 g of methanol, and 9.88 g of p-toluenesulfonic acid monohydrate were added. The mixture was stirred and reacted at room temperature for 30 min, and the system became clear and transparent. An amount of 15.00 g of NaHCO_3_ was added for neutralization, and insoluble substances were removed by filtration. The methanol was evaporated to dryness under reduced pressure at 50 °C. An amount of 150 mL of ethyl acetate and 100 mL of distilled water were added, and the mixture was separated. Then, 100 mL of distilled water was added to wash the ethyl acetate. The ethyl acetate was evaporated to dryness, yielding 24.90 g of white solid HMMM (yield rate 83%). The masses of the reagents were measured using an analytical balance with a readability of 0.01 g, and the reaction temperature was maintained within ±1 °C of the set temperature.

### 2.6. Curing of Tetra-Component Phenolic Resin (Scheme 4)

A 30% solution was prepared by dissolving 10.00 g of the tetra-component phenolic resin in 24.00 g of PGMEA. Subsequently, 0.038 g of the catalyst, p-toluenesulfonic acid monohydrate, was added to a 1.00 g aliquot of this solution. The mixture was heated to 80 °C to dissolve the catalyst. Then, 0.060 g of the crosslinking agent HMMM was added. The mixture was heated to 90 °C. After magnetic stirring for 10 min, the mixture stopped flowing and completely gelled into a brown, jelly-like elastic substance. Optimization of the parameters which affect the curing of tetra-component phenolic resin is summarized in Table 2. The tetra-component phenolic resin, PGMEA, and the resin solution aliquot were weighed using an electronic balance with a readability of 0.01 g, while p-toluenesulfonic acid monohydrate and HMMM were weighed using an analytical balance with a readability of 0.0001 g. And the reaction temperature was maintained within ±1 °C of the set temperature.

**Scheme 4:**
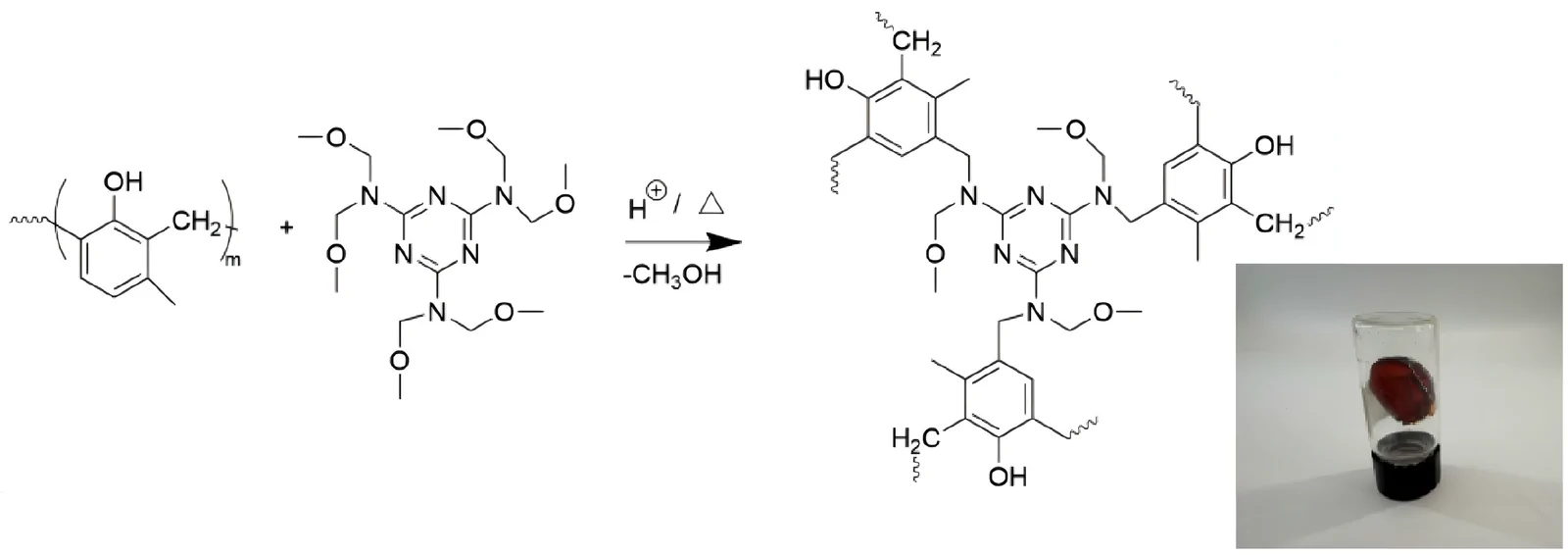
The curing mechanism of tetra-component phenolic resin and the picture of elastomer real products.

### 2.7. Testing and Characterization

FTIR test: Spectra were recorded over a scanning range of 4000 to 400 cm^−1^ at a resolution of 0.5 cm^−1^.

NMR test: Both ^1^H NMR and ^13^C NMR spectra were acquired using deuterated acetone as the solvent, with 16 and 256 scans, respectively.

GPC test: Molecular weight distribution was determined using a PLgel MIXED-E tandem column with tetrahydrofuran as the mobile phase and a differential refractive index detector.

DSC test: DSC was used to thermally characterize the samples using a heating/cooling rate of 10 °C·min^−1^.

Film thickness measurement: The resin was dissolved in PGMEA to form a 30 wt% solution, which was then spin-coated onto a 2-inch silicon wafer and dried. The film thickness was subsequently measured using an F20 thin-film thickness measurement instrument.

## 3. Results and Discussion

**Scheme 5:**
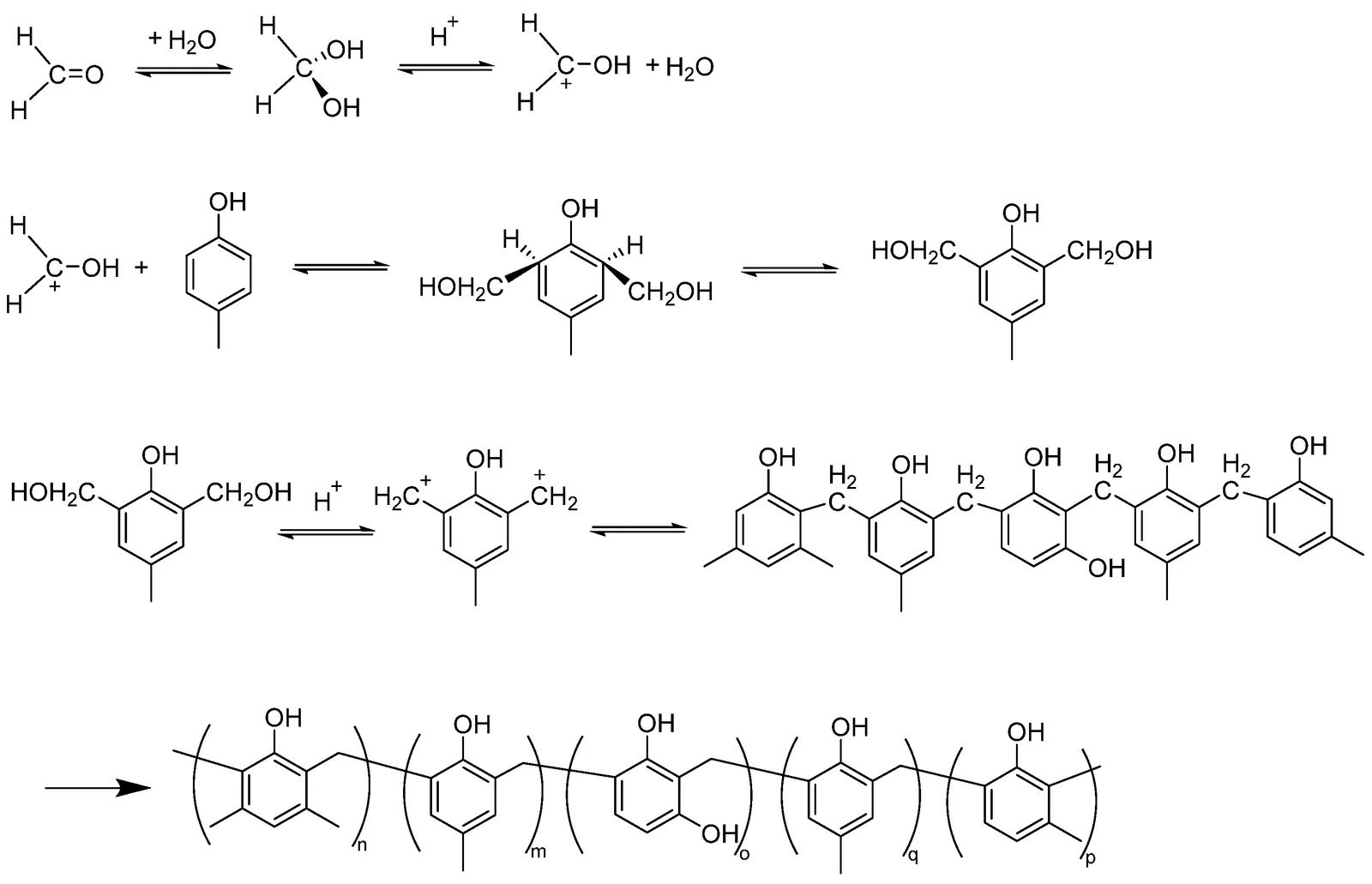
The synthetic mechanism of tetra-component phenolic resin.

The acid-catalyzed polymerization process of tetra-component phenolic resin (Scheme 5).

### 3.1. FTIR Analysis

**Figure 1:**
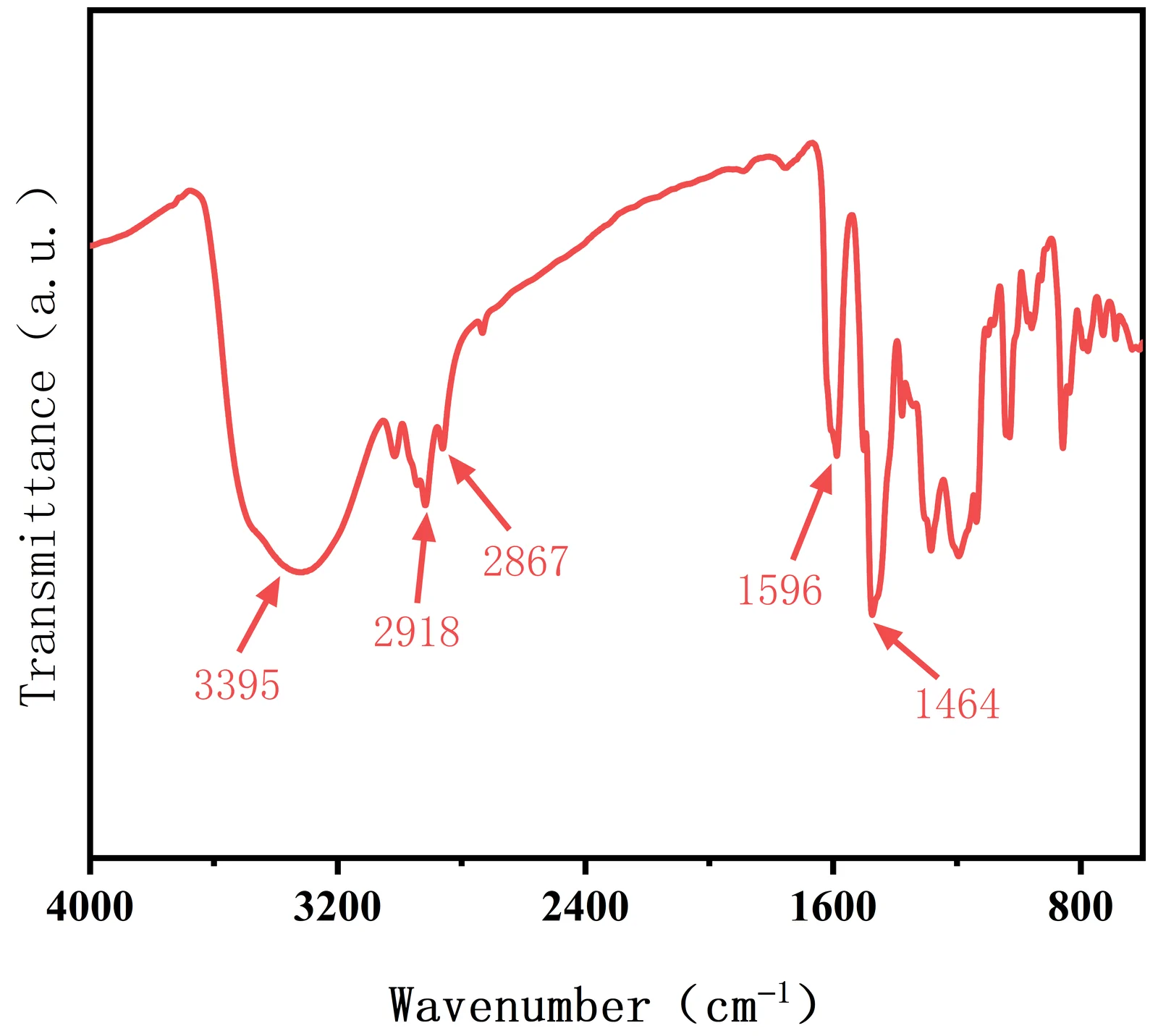
FTIR of tetra-component phenolic resin.

Successful preparation of the polymer was indicated by the FTIR study of the tetra-component phenolic resin. The FTIR spectrum of the tetra-component phenolic resin is presented in Figure 1. The absorption band at 3395 cm^−1^ was assigned to O–H stretching, while the peaks at 2918 and 2867 cm^−1^ arise from C–H stretching of methyl and methylene groups. The absorptions at 1596 and 1464 cm^−1^ were characteristic of the benzene ring skeleton. All of these peaks confirm that the tetra-component phenolic resin was successfully synthesized.

### 3.2. ^1^H NMR Analysis

**Figure 2:**
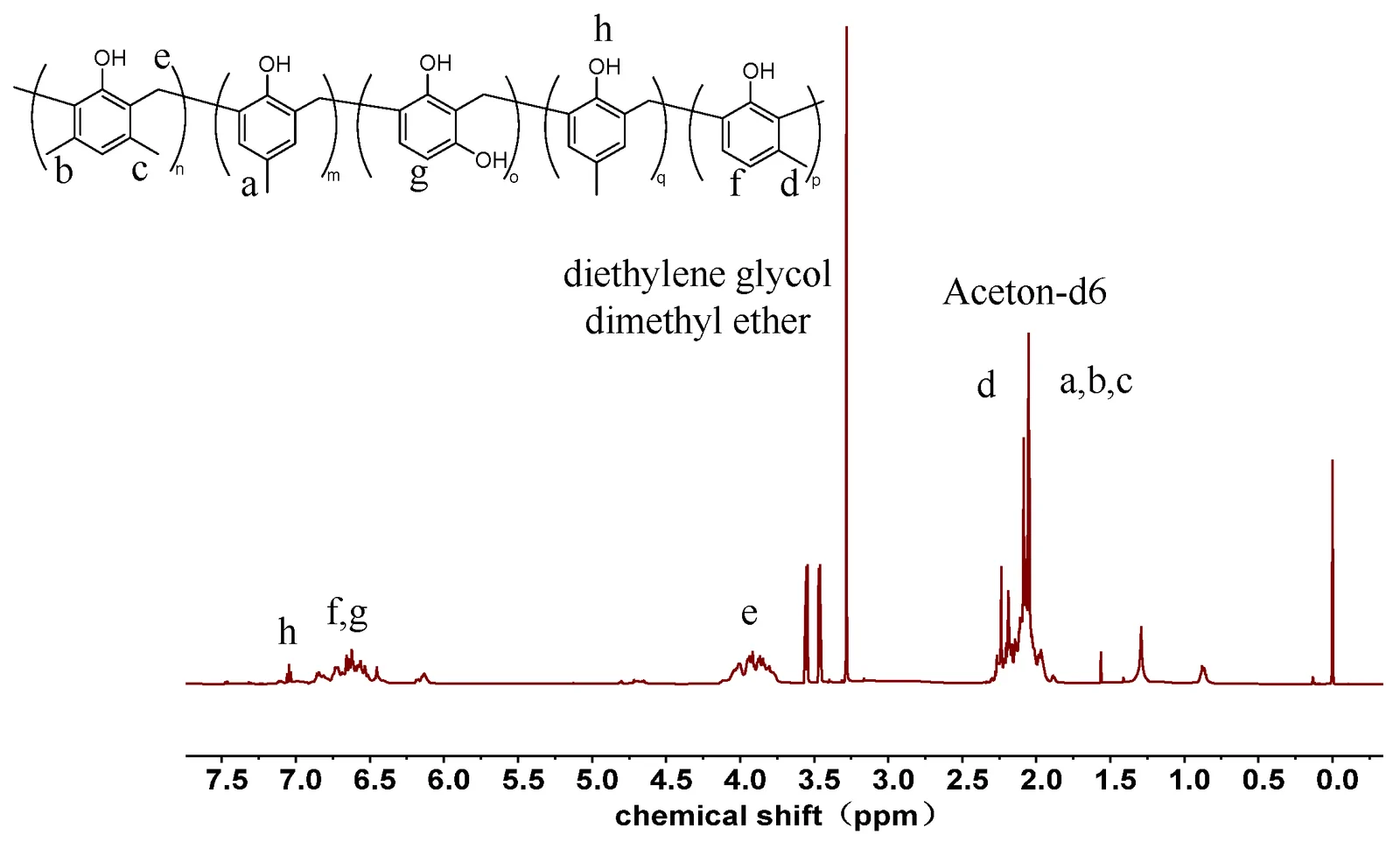
^1^H NMR of tetra-component phenolic resin.

The ^1^H NMR spectrum of the resin is shown in Figure 2. The chemical shift at δ 7.1 is attributable to the hydroxyl proton (denoted h). The aromatic protons (denoted f, g) appeared as multiple signals in the region of δ 6.40–6.90. The resonancesobserved between δ 3.70 and 4.10 correspond to the methylene bridges (–CH_2_–) (denoted e) connecting the aromatic rings. The methyl protons (denoted a, b, c and d) gave rise to signals in the range of δ 1.50–2.20. The solvent peak for deuterated acetone was observed at δ 2.05. So, the ^1^H NMR spectra support the formation of the tetra-component phenolic resin. 

### 3.3. ^13^C NMR Analysis

**Figure 3:**
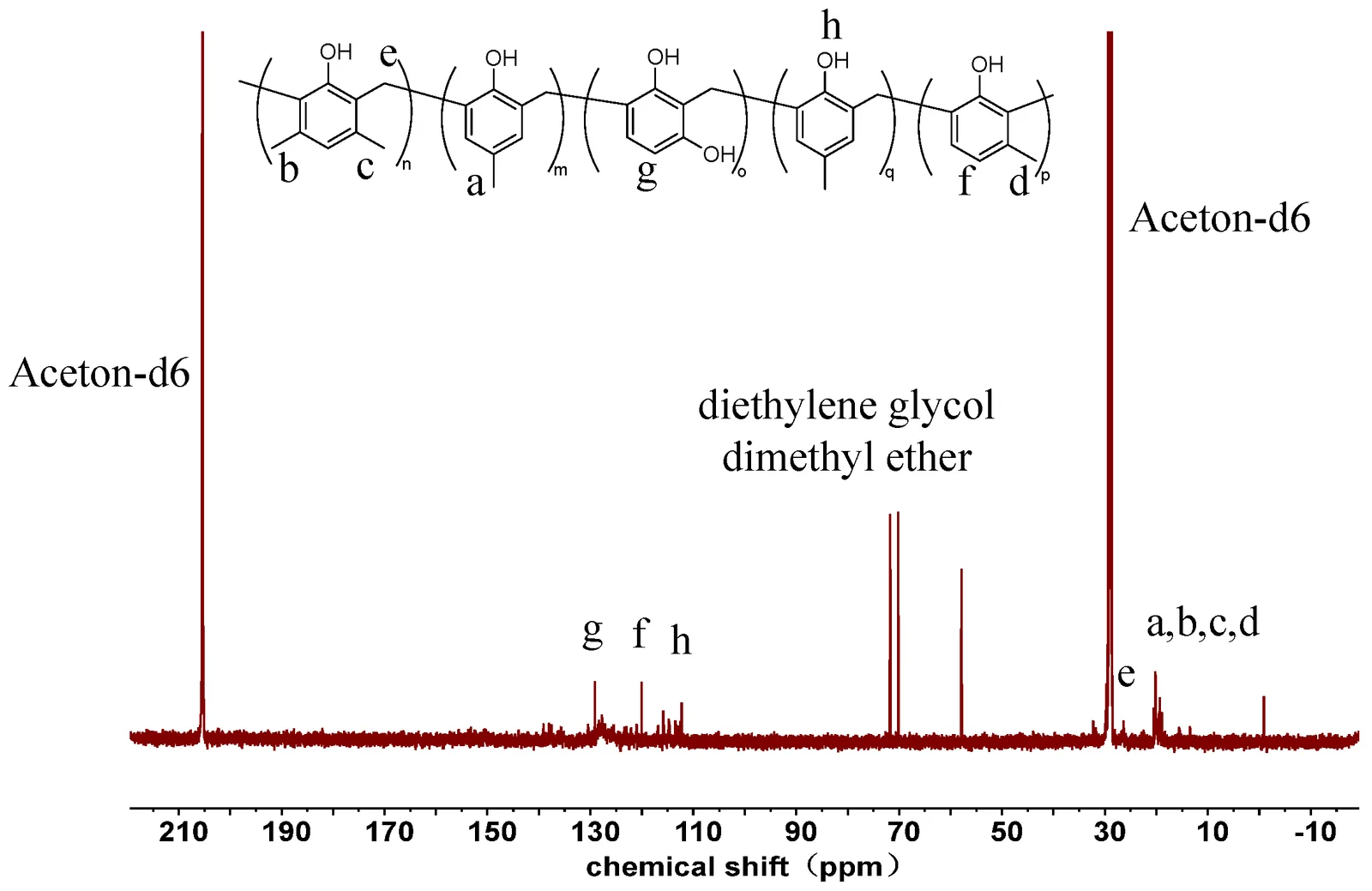
^13^C NMR of tetra-component phenolic resin.

In the ^13^C NMR spectrum (Figure 3), the signals in the range of δ 110–130 were assigned to the aromatic carbon atoms (denoted g, f and h). The resonance at δ 26 corresponds to the methylene carbon at position e, while the signals between δ 19 and 21 are attributed to the methyl carbons at positions a, b, c, and d. The peaks at δ 210 and 30 arise from the deuterated acetone solvent. So, the ^13^C NMR spectra support the formation of the tetra-component phenolic resin.

### 3.4. GPC Analysis

**Figure 4:**
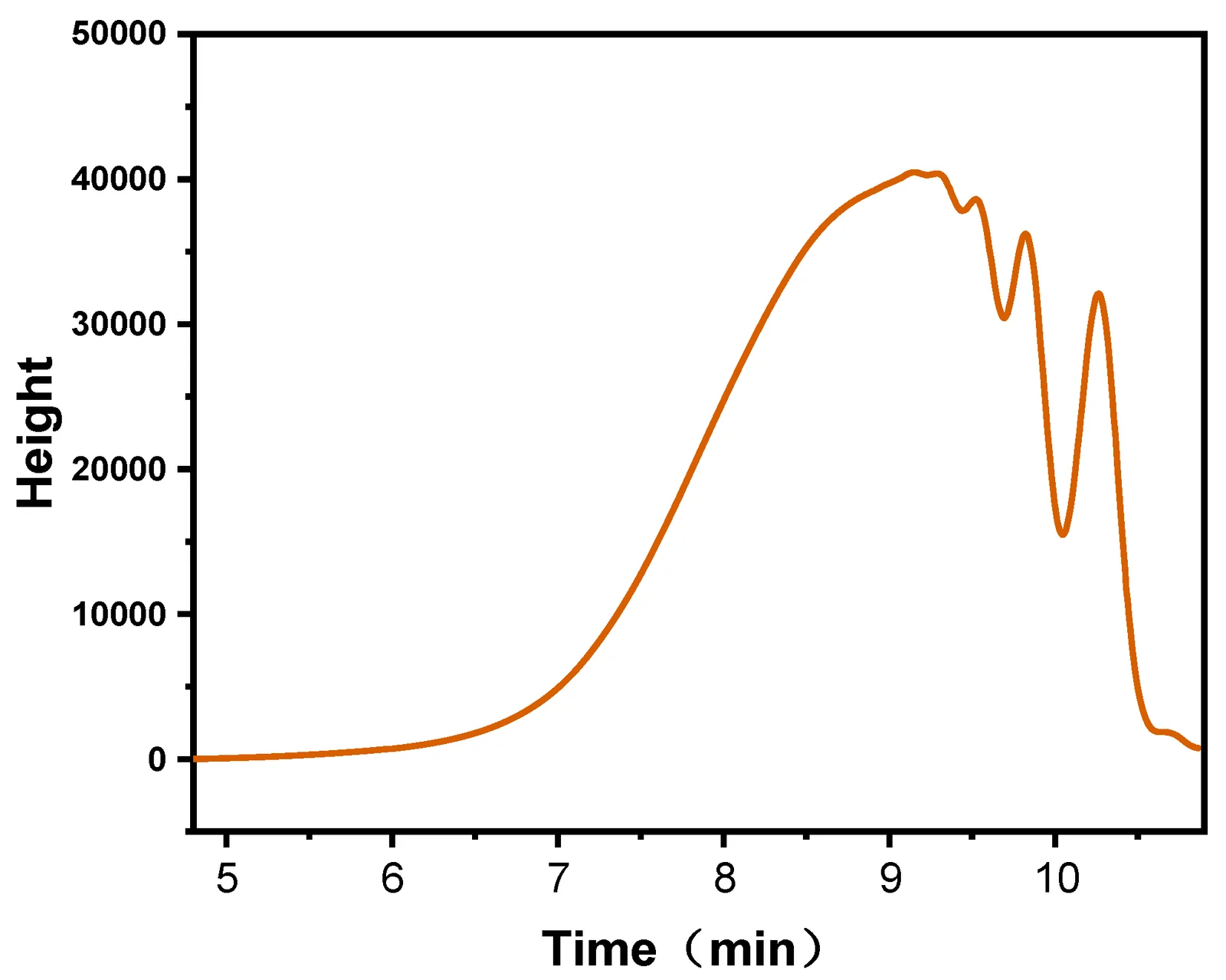
GPC of tetra-component phenolic resin.

The GPC curve of the tetra-component phenolic resin exhibited a unimodal distribution, indicating a relatively uniform molecular weight (Figure 4). GPC experimental method: The calibration standard is narrow-distribution polystyrene with a calibration range of 580–48,300 g/mol, a flow rate of 1 mL/min, and a column temperature of 40 °C. The sample is dissolved in chromatographic-grade THF to form a 5 mg/mL solution for testing. *M*_n_ and *M*_w_ are usually “polystyrene conversion values” and do not represent absolute molecular weight.

Due to the ordinary g/i line DNQ novolac photoresist resin, *M*_n_ ≈ 1500–2500, *M*_w_ ≈ 3500–12,000, and dispersion ≈ 1.5–3 are often used as the performance balanced range. Four-component phenolic resin *M*_n_ (the number-average molecular weight) = 3520 g/mol, *M*_w_ (the weight-average molecular weight) = 6376 g/mol, and dispersity (PDI = *M*_w_/Mn) = 1.81, which preliminarily meets the film-forming requirements for photoresist applications [27]. These results confirm the successful synthesis of the tetra-component phenolic resin.

### 3.5. Comparison of DSC

**Figure 5:**
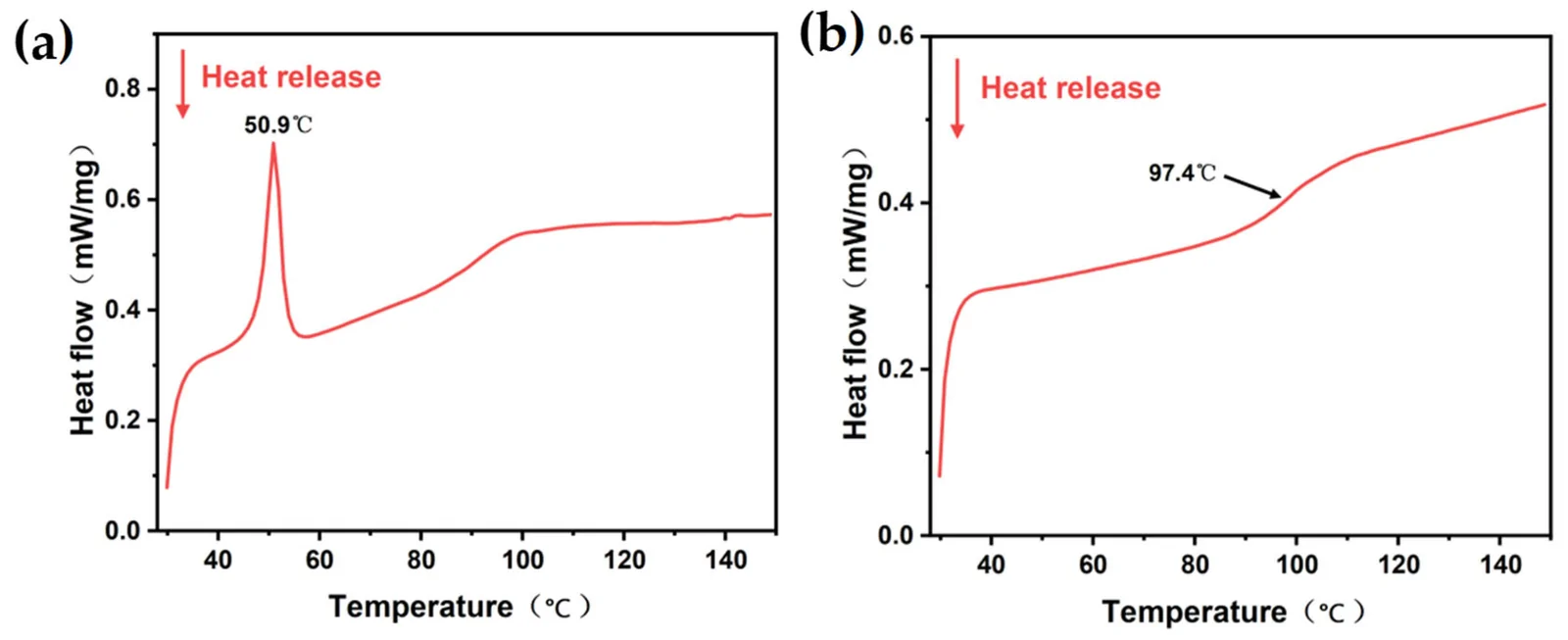
DSC curves of (**a**) the first heating scan and (**b**) the second heating scan of the mixture of tetra-component phenolic resin, catalyst, and crosslinking agent.

The sample for the DSC test was the mixture of tetra-component phenolic resin (0.3 g), catalyst p-toluenesulfonic acid monohydrate (0.038 g), and crosslinking agent HMMM (0.06 g). DSC experimental method: atmosphere: high-purity nitrogen gas; gas flow rate: 40 mL/min; sample mass: 5.82 mg; complete temperature range: 30 °C to 150 °C; heating rate: 10.0 K/min; and temperature history between the first and second scans: cooling from 150 °C to 30 °C. The formula used in the DSC experiment is the same as Entry 5 in Section 2.6, except that there is no solvent PGMEA. Figure 5a is the first heating scan. As can be seen, an endothermic peak is observed around 50.9 °C, corresponding to the melting of HMMM. Figure 5b is the second heating scan. As can be seen, a step is observed around 97.4 °C, corresponding to the *T*_g_ of the elastomer. The DSC results are consistent with the changes related to the curing/crosslinking process.

### 3.6. Alkaline Solubility Evaluation (ADR)

ADR refers to the speed at which a material dissolves in an alkaline solution, and it holds significant importance, particularly in the applications of photoresists and phenolic resins. A 30 wt% solution of the tetra-component phenolic resin was prepared by dissolving the resin in PGMEA. This solution was then spin-coated onto a silicon wafer surface and baked at 100 °C for 3 min to remove volatile solvents, yielding a photoresist film. The film thickness was controlled within the range of 3~4 μm and measured using a film thickness meter, denoted as δ. The film was subsequently immersed in a 2.38 wt% tetramethylammonium hydroxide (TMAH) aqueous solution, and the time required for complete dissolution was recorded as t. The ADR of the tetra-component phenolic resin was calculated as follows:

ADR = δ/t

Table 3 contains multiple thickness measurements for each test, which are the measurement results for different locations on the same film. The pure resin used for DNQ/positive novolac photoresist has an ADR value of 159 ± 2.16 Å/s (three independent experiments) under the conditions of 23 °C and 2.38 wt% TMAH. Because the reference range provided by common technical literature [33] is approximately 50–200 Å/s for ADR of pure resins used in high-resolution formulations, the ADR value of 159 ± 2.16 Å/s is considered a “good” ADR for pure resins. The tetra-component phenolic resin exhibited an average ADR of 159 ± 2.16 Å/s, indicating favorable solubility in an alkaline developer and thus demonstrating its potential for further investigation in photoresist formulation.

## 4. Conclusions

The tetra-component phenolic resin reported in this study was synthesized via addition, condensation, and polycondensation reactions of 2,6-bis(hydroxymethyl)-4-methylphenol, 3,5-dimethylphenol, resorcinol, and m-cresol. GPC analysis revealed a weight-average *M*_w_ of 6376 g/mol, and the resin exhibited an ADR of 159 ± 2.16 Å/s. The ADR plays a crucial role in the production and application of photoresists. Through rational experimental design and controlled conditions, ADR can be effectively measured and optimized to meet the needs of different applications. The main experimentally determined properties (e.g., Mw, softening point, and ADR) of the present resin, conventional novolac, and structurally controlled novolac are compared in Table 4. Due to the ordinary g/i line DNQ novolac photoresist resin, softening point ≈ 130–160 °C and ADR ≈ 100–1600 Å/s are often used as the performance-balanced range. Four-component phenolic resin, softening point = 135 °C, and ADR = 159 ± 2.16 Å/s preliminarily meet the film-forming requirements for photoresist applications. These results demonstrate that the tetra-component phenolic resin preliminarily meets the requirements for photoresist film formation and could have the opportunity to be used as a candidate resin for further investigation in photoresist formulations. Due to limitations in conditions, the application of the resin produced in photoresist formulation was not discussed in detail in the article.

## Figures and Tables

**Table 1:** Evaluation of the selected reaction conditions which affect the synthesis of tetra-component phenolic resin.

Entry	Temp (°C)	Time (h)	Solvent	*M*_w_ (g/mol)	Softening Point (°C)
1	100	18	diethylene glycol dimethyl ether	6376	135
2	90	18	diethylene glycol dimethyl ether	5410	123
3	100	12	diethylene glycol dimethyl ether	6115	128

**Table 2:** Optimization of the curing of tetra-component phenolic resin.

Entry	PTSA·H_2_O (g)	HMMM (g)	Temp (°C)	Time (min)	Sample Status
1	0.038	0.012	90	10	liquid
2	0.038	0.024	90	10	liquid
3	0.038	0.036	90	10	liquid
4	0.038	0.048	90	10	liquid
5	0.038	0.060	90	10	gel

**Table 3:** ADR of tetra-component phenolic resin.

Film Thickness (Å)			Developing Time (s)	ADR (Å/s)
31,752	31,669	31,719	201	158
30,905	30,840	30,686	196	157
30,834	30,339	30,292	188	162

**Table 4:** Comparison of the main experimentally determined properties of the present resin, conventional, and structurally controlled novolac.

Resin	M/P/XY *	*M*_w_ (g/mol)	Softening Point (°C)	ADR (Å/s)
Conventional novolac	50/50/0	7600–9200	140–170	270–330
Structurally controlled novolac	45/35/20	6500–7500	150–170	110–130
Tetra-component phenolic resin	/	6376	135	159 ± 2.16

* M/P/XY represents m-cresol/p-cresol/xylenol.

## Data Availability

The original contributions presented in this study are included in the article. Further inquiries can be directed to the corresponding authors.

## Abbreviations

LCD	Liquid crystal display
DNQ	Diazonaphthoquinone
FTIR	Fourier transform infrared
NMR	Nuclear magnetic resonance
GPC	Gel permeation chromatography
HMMM	Hexamethoxymethylmelamine
PGMEA	Propylene glycol monomethyl ether acetate
M_w_	Molecular weight
ADR	Alkaline dissolution rate
TMAH	Tetramethylammonium hydroxide
*T*_g_	Glass transition temperature
DSC	Differential scanning calorimetry
PTSA·H_2_O	*p*-Toluenesulfonic acid monohydrate
